# Supplementary material for: Ecological Diversification in an Adaptive Radiation of Plants: The Role of De Novo Mutation and Introgression
Source: Mol Biol Evol. 2024 Jan 17;41(1):msae007. doi: 10.1093/molbev/msae007 (PMC10826641; doi:10.1093/molbev/msae007)
Supplement: msae007_Supplementary_Data [file msae007_supplementary_data.pdf]

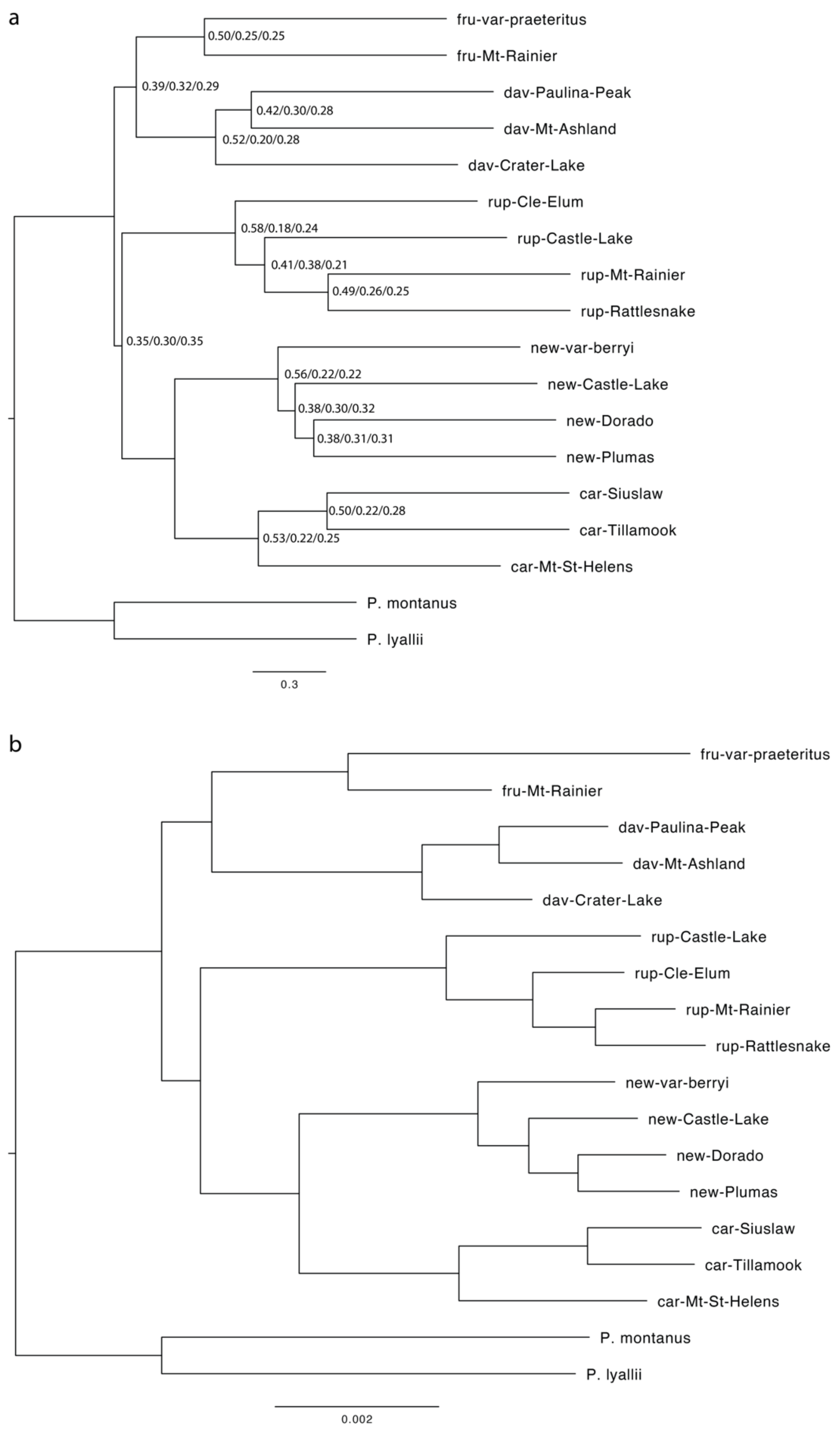

Figure S.1. Alternative species trees. (a) ASTRAL CDS tree. Labels at nodes indicate ASTRAL quartet scores for q1, q2, and q3, respectively. All ASTRAL local posterior probabilities are 1. (b) Concatenated MLE tree. All UFBoot values are 100%.

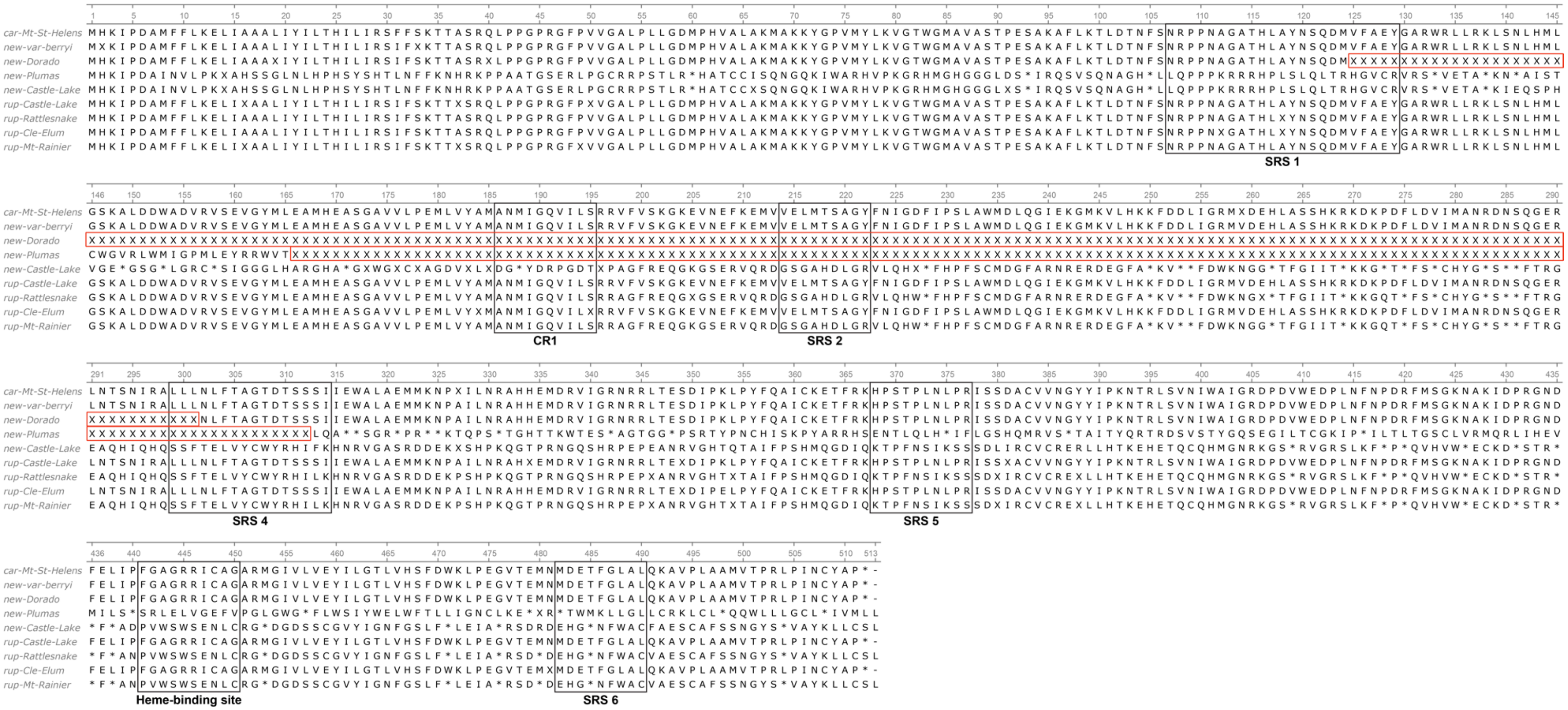

Figure S.2. Protein alignment of *F3'5'h* for a reference *P. cardwellii* (known functional copy) and all samples of *P. newberryi* and *P. rupicola*. Black boxes outline substrate recognition sites (SRS), the heme-binding domain, and the conserved hydroxylation activity site (CR1). We were unable to definitively identify the position of SRS3 through sequence homology. Red boxes outline large deletions in two of the *P. newberryi* samples.

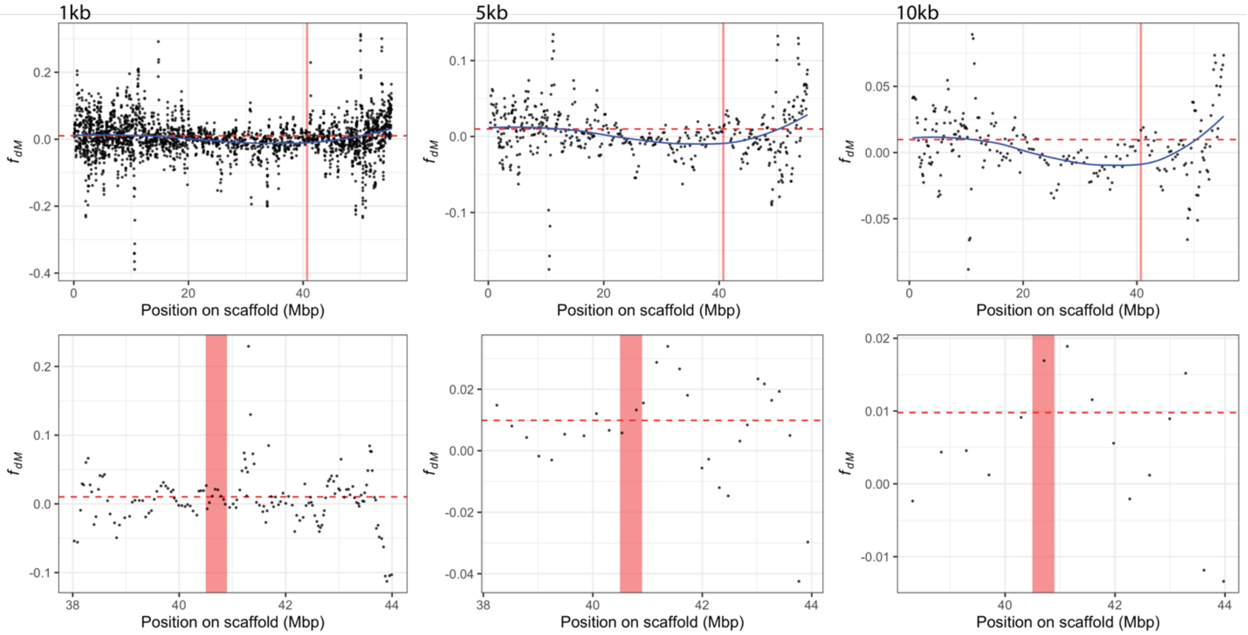

Figure S.3. Values of f_{dM} in non-overlapping windows surrounding the *F3'5'h* locus. Window sizes are 1,000 SNPs, sliding every 250 SNPs (left), 5,000 SNPs, sliding every 1,250 SNPs, (center), and 10,000 SNPs, sliding every 2,500 SNPs (right).

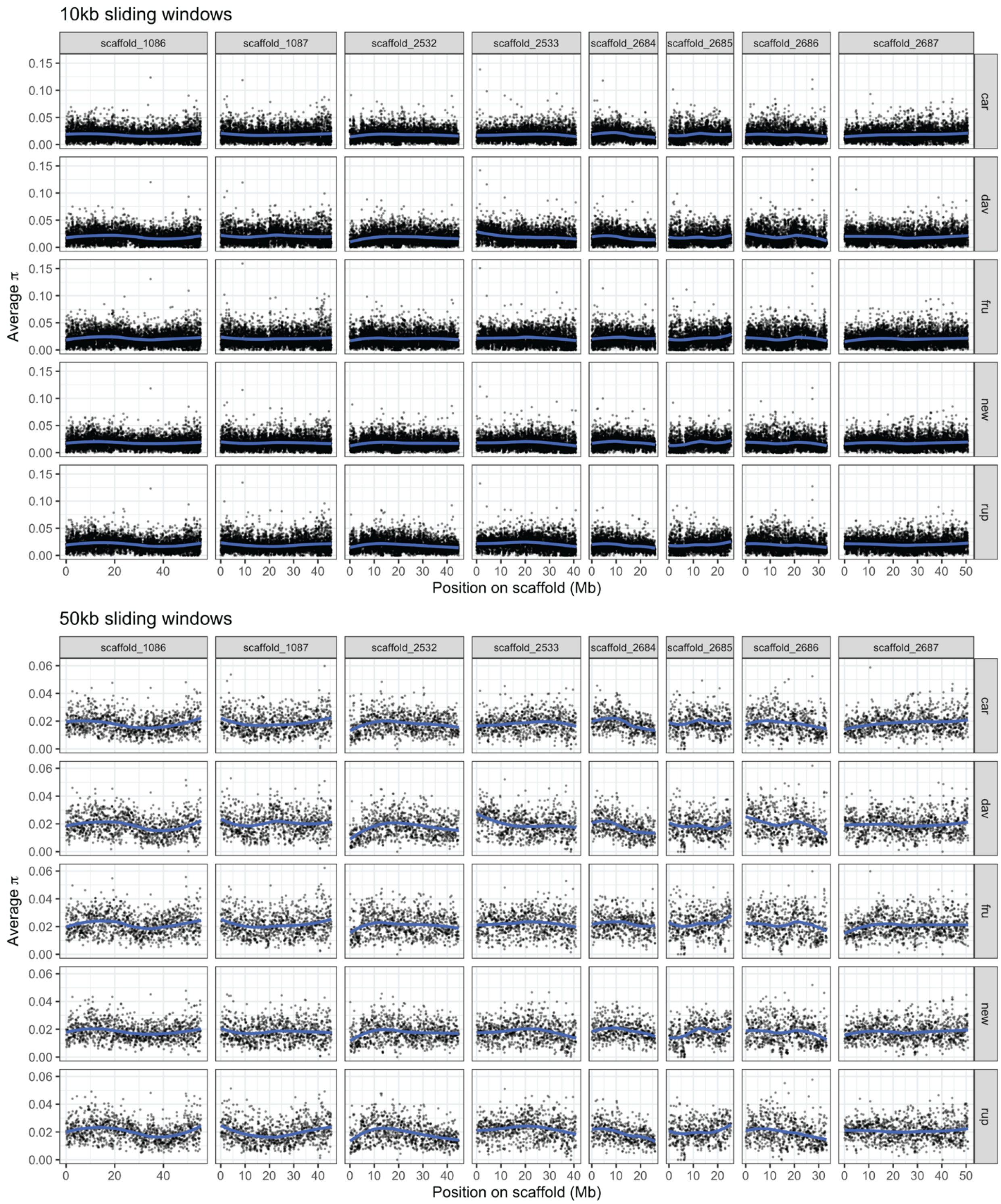

Figure S.4. Values of π across the eight main scaffolds of the *P. davidsonii* reference genome in 10kb (top) and 50kb (bottom) windows.

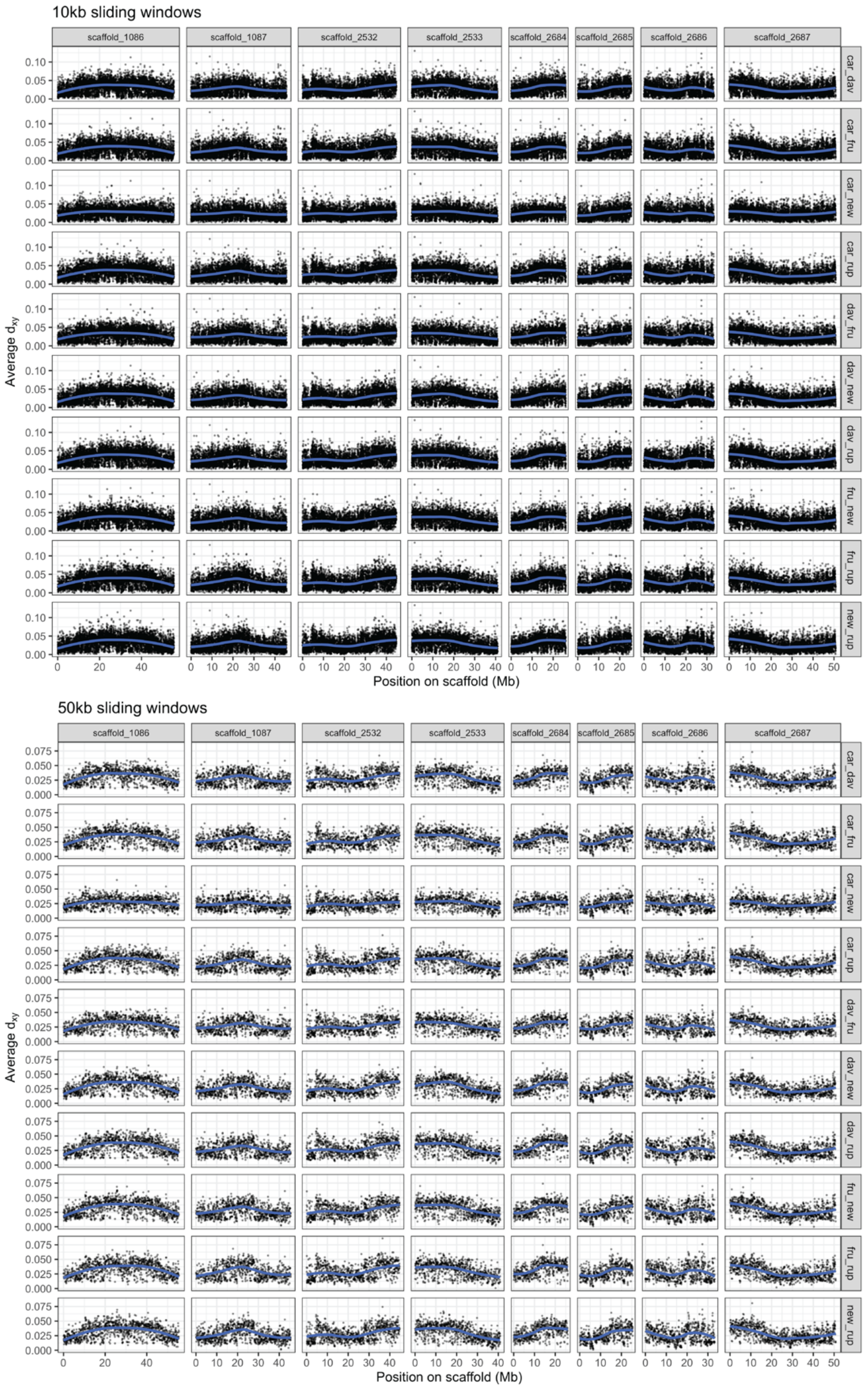

Figure S.5. Values of d_{xy} across the eight main scaffolds of the *P. davidsonii* reference genome in 10kb (top) and 50kb (bottom) windows.

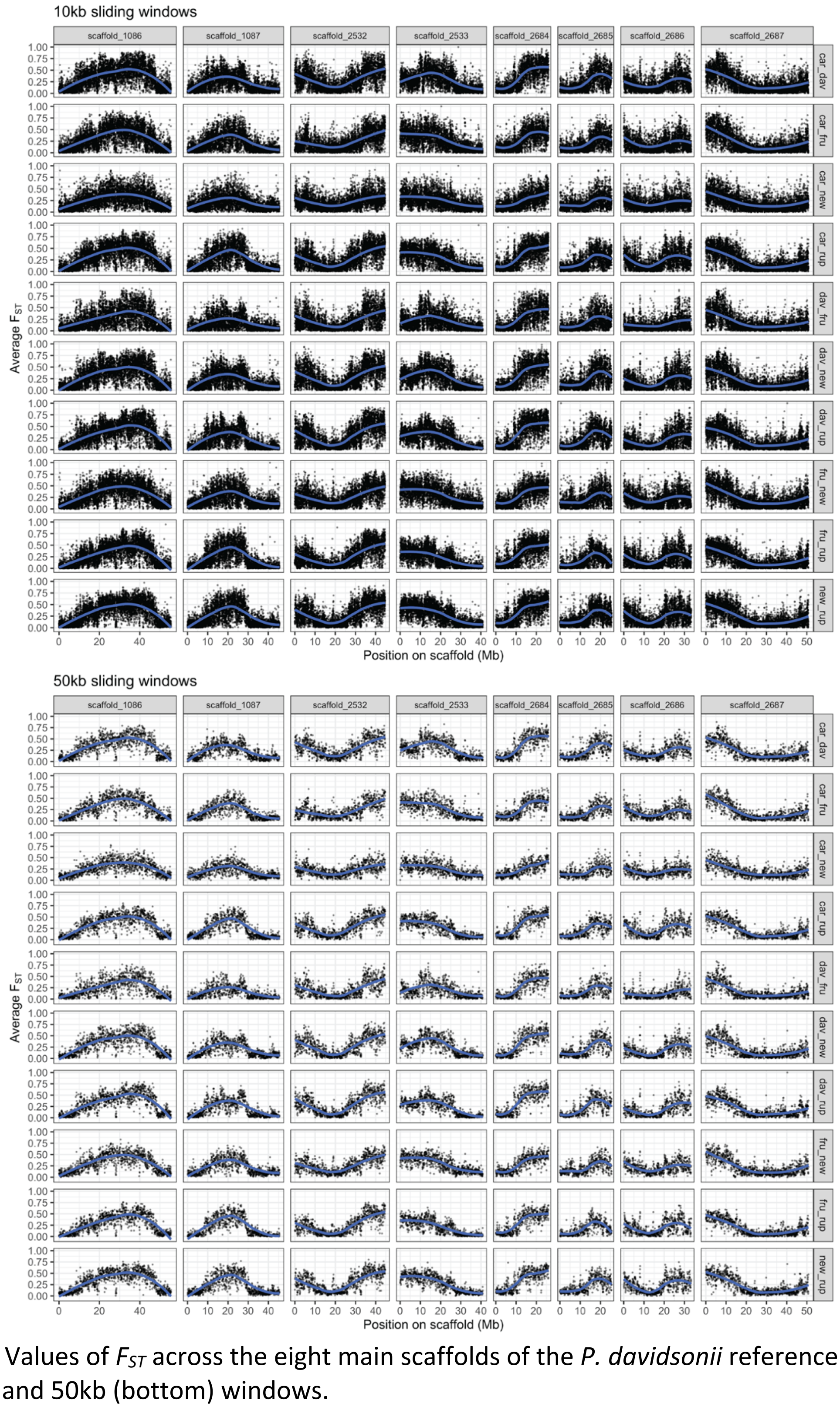

Figure S.6. Values of F_{ST} across the eight main scaffolds of the *P. davidsonii* reference genome in 10kb (top) and 50kb (bottom) windows.

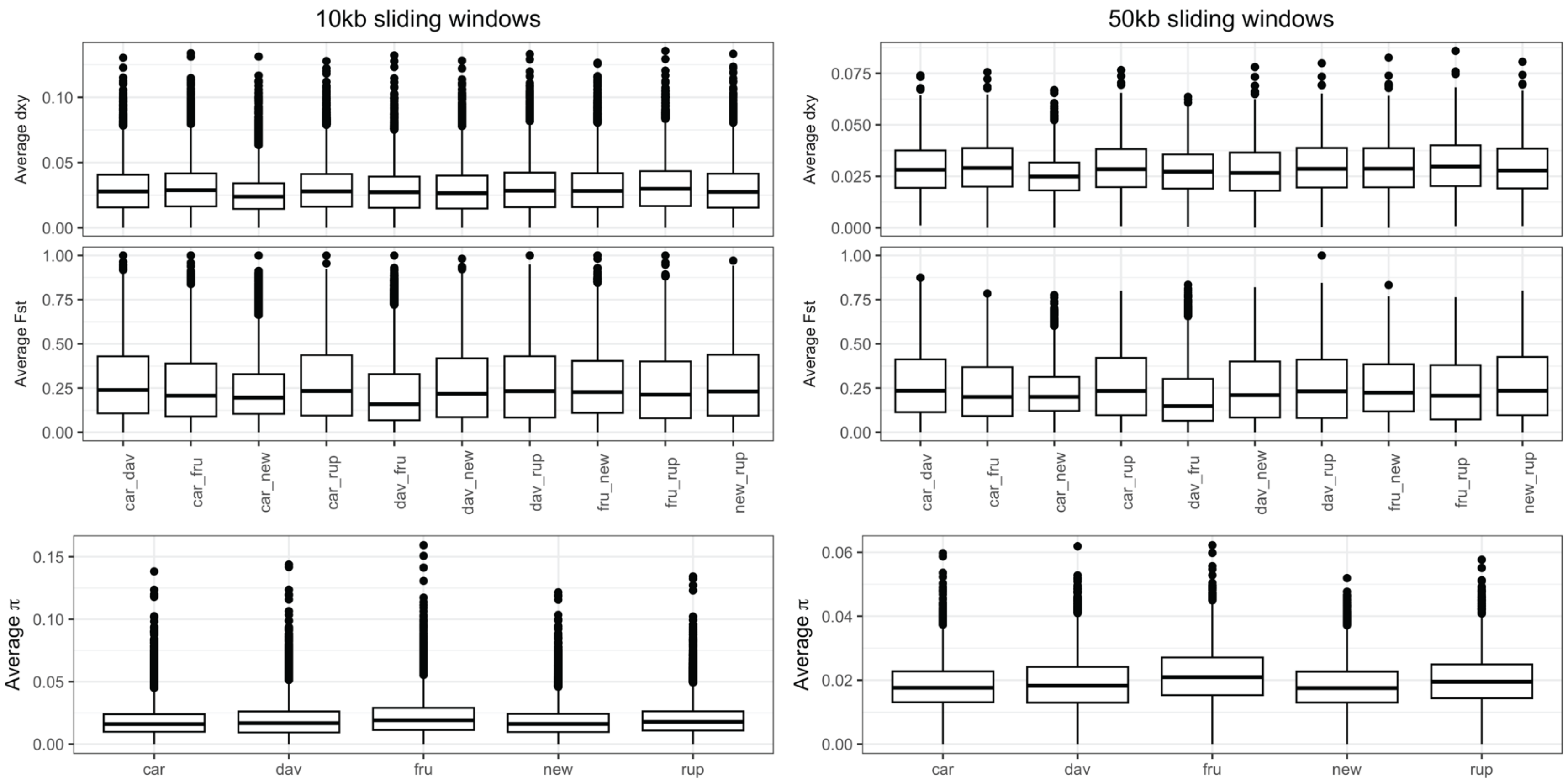

Figure S.7. Genome-wide values of d_{xy}, F_{ST}, and π in 10kb (left) and 50kb (right) windows.

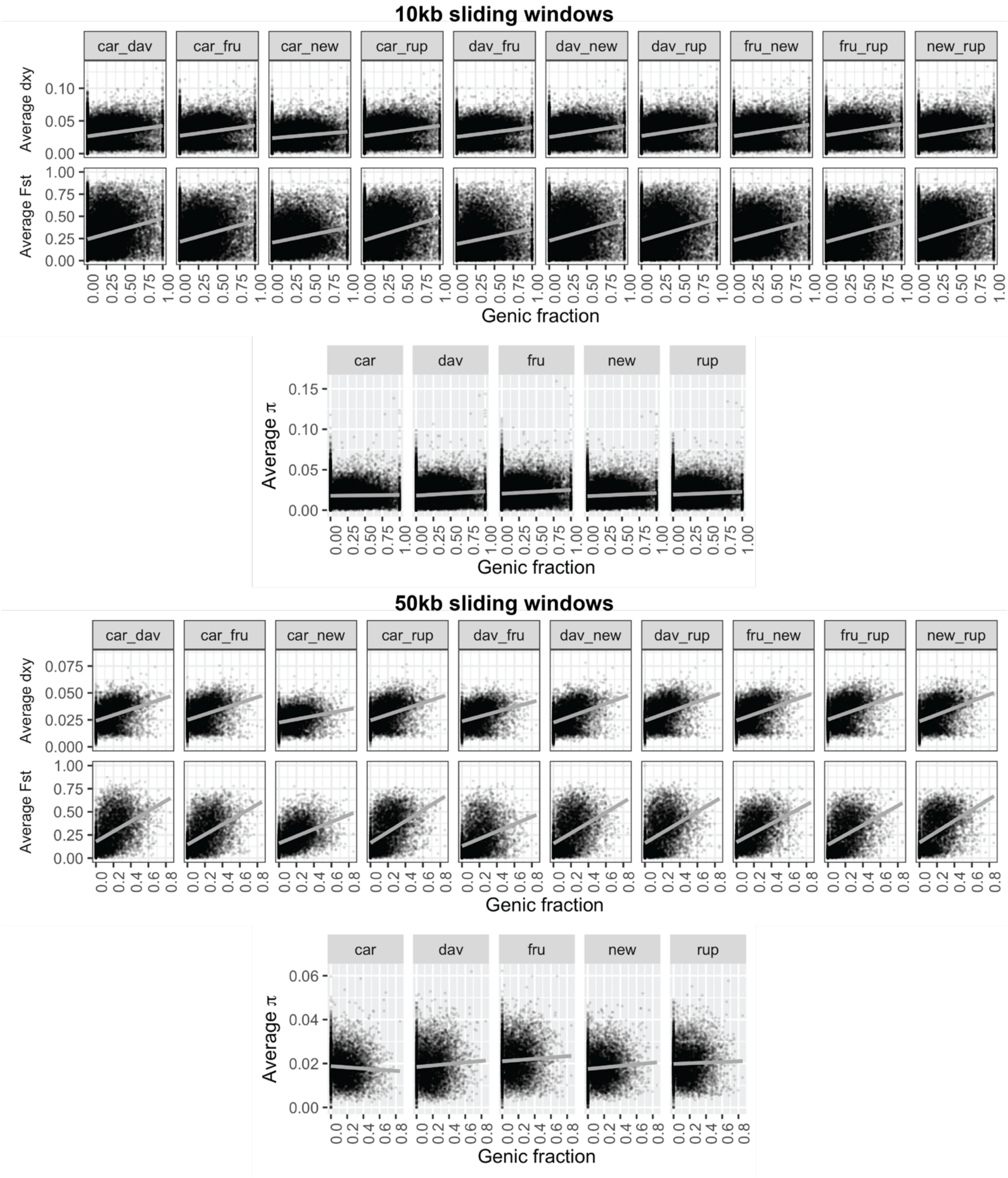

Figure S.8. Linear regressions between genic fraction and diversity and divergence metrics (d_{xy}, F_{ST}, and π) in 10kb (top) and 50kb (bottom) windows.

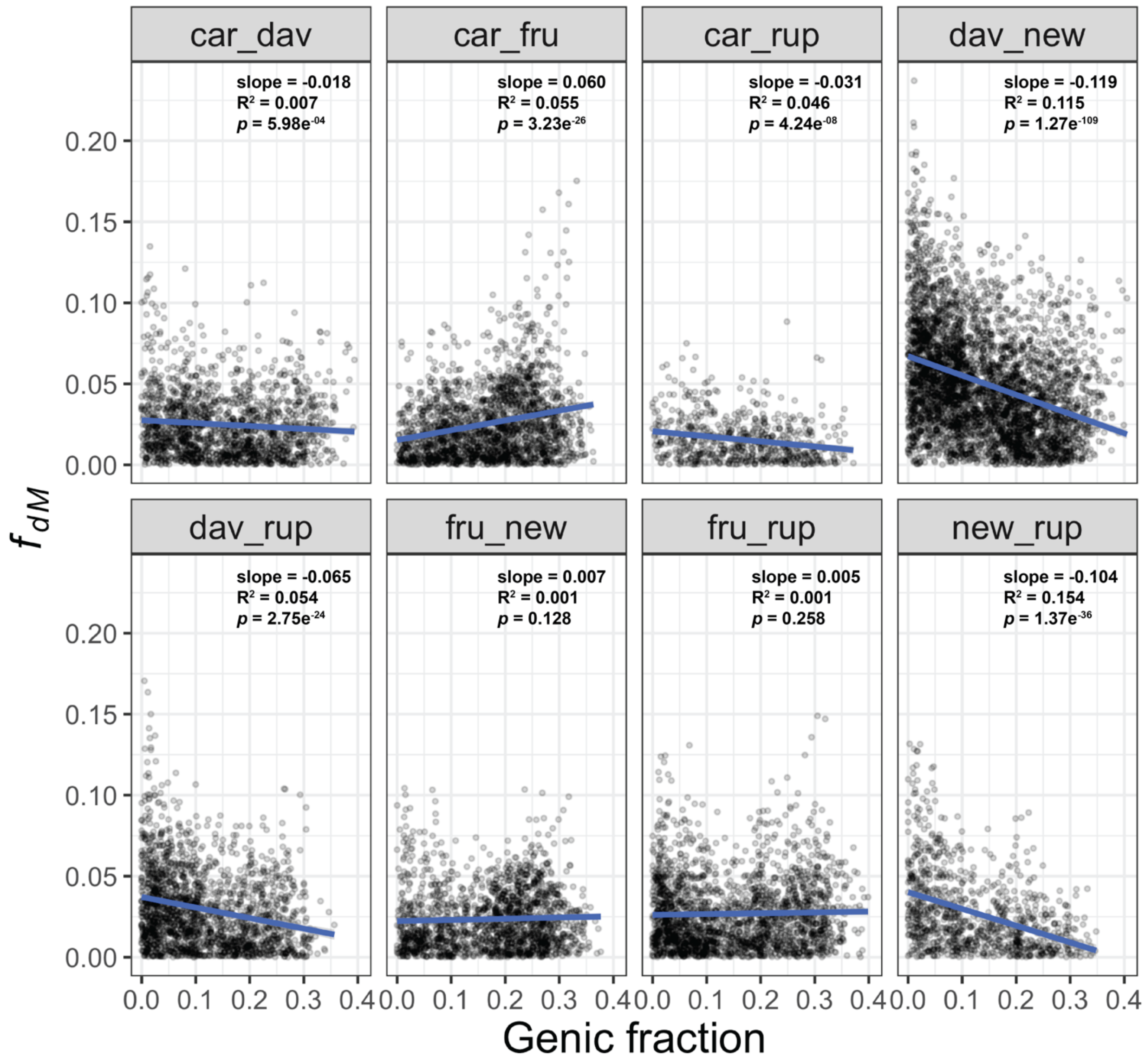

Figure S.9. Linear regressions between genic fraction and introgression (f_{dM}). f_{dM} was calculated in 10,000 SNP windows, sliding every 2,500 SNPs.

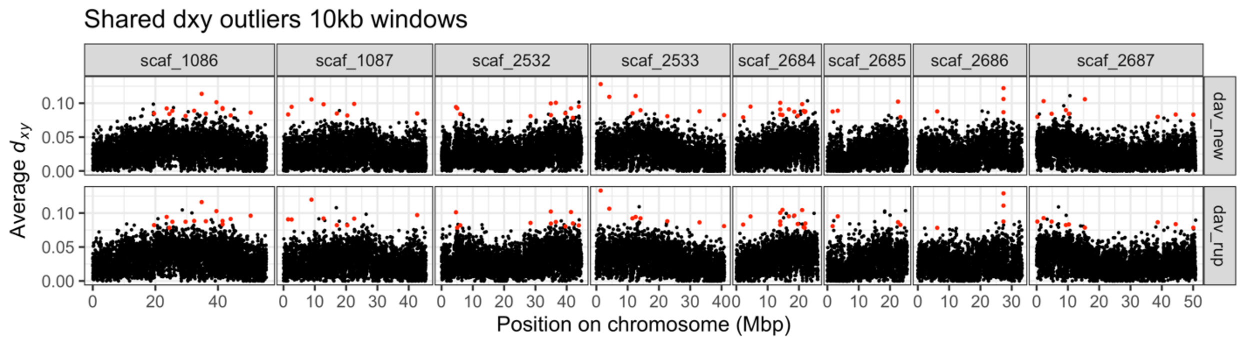

Figure S.10. d_{xy} in sliding 10kb windows in two species pairs known to form hybrid zones (*P. davidsonii-P. newberryi* and *P. davidsonii-P. rupicola*). Outlier d_{xy} values (Z-score >3) in the same windows for both species pairs are highlighted in red.

Table S.1. Sampling information

Species	Identifier	Population ID	Latitude	Longitude
Penstemon rupicola	Castle Lake	86-8	41.223113	-122.379630
	Rattlesnake	98-4	47.436029	-121.778519
	Mt. Rainier	105-2	46.778033	-121.760309
	Cle Elum	101-1	47.411009	-121.092183
Penstemon davidsonii	Mt. Ashland	87-7	42.081020	-122.718583
	Crater Lake	116-7	42.895602	-122.092252
	Paulina Peak	117-2	43.684535	-121.265441
Penstemon newberryi	Dorado	75-9	38.661708	-120.130671
	Plumas	80-5	39.880031	-121.161161
	var. *berryi*	84-4	41.389587	-122.993862
	Castle Lake	85-9	41.231111	-122.382305
Penstemon cardwellii	Mt. St. Helens	28-5	46.313721	-122.036324
	Siuslaw	91-6	44.274483	-123.579297
	Tillamook	92-7	45.210899	-123.758863
Penstemon fruticosus	Mt. Rainier	106-1	46.768161	-121.674510
	var. *praeteritus*	118-1	42.735390	-118.633691
Penstemon lyallii	Kaniksu	44-9	48.371445	-116.156739
Penstemon montanus	Challis	61-7	44.511154	-114.374046

Table S.2. Read depth and coverage information.

			**Scaffolds**	**1086**	**2687**	**1087**	**2532**	**2533**	**2686**	**2684**	**2685**	1085	2151	2531	2446		
Species	Sample	reads mapped and paired	mean genome-wide depth	6E+07	5E+07	5E+07	4E+07	4E+07	3E+07	3E+07	3E+07	1E+07	8E+06	7E+06	5E+06	Total bp	Total coverage
Penstemon rupicola	rup-Castle-Lake	26629396	11.8227	77.97%	73.11%	72.59%	74%	76.15%	71.90%	76.89%	71.23%	68.58%	70.92%	60.71%	64.40%	260102573	0.737
	rup-Rattlesnake	32852064	14.0107	77.59%	71.70%	72.79%	74.55%	74.69%	70.36%	76.98%	68.43%	68.18%	72.45%	53.78%	68.60%	257684226	0.730
	rup-Mt-Rainier	24422232	11.1638	77.89%	71.17%	71.71%	73.31%	74.72%	70.30%	76.71%	68.05%	68.18%	68.89%	57.75%	67.24%	256275894	0.726
	rup-Cle-Elum	26171512	11.5766	78.36%	73.43%	71.91%	75.00%	76.17%	70.33%	76.50%	68.79%	67.67%	71.33%	52.55%	64.14%	258900747	0.734
Penstemon davidsonii	dav-Mt-Ashland	27095921	11.8077	81.61%	74.60%	73.67%	76.73%	78.28%	75.14%	80.83%	71.97%	70.01%	71.16%	58.86%	74.50%	268470978	0.761
	dav-Crater-Lake	29184105	12.0241	81.39%	75.15%	74.09%	76.72%	76.87%	72.41%	80.67%	71.86%	70.37%	72.43%	56.92%	72.82%	267182235	0.757
	dav-Paulina-Peak	31564339	13.1641	81.71%	75.01%	73.93%	76.37%	77.35%	75.46%	80.42%	71.87%	71.61%	69.99%	58.10%	74.93%	268339775	0.760
Penstemon newberryi	new-Dorado	40276056	13.8701	81.33%	76.65%	77.14%	76.90%	79.13%	75.47%	80.06%	76.51%	72.38%	74.76%	67.81%	68.57%	273259508	0.774
	new-Plumas	36370992	15.2172	80.53%	75.92%	76.74%	76.40%	78.85%	73.32%	79.72%	75.46%	70.27%	74.60%	68.12%	68.86%	270644978	0.767
	new-var-*berryi*	29458522	12.2794	79.83%	74.39%	75.24%	75.54%	77.15%	74.28%	78.91%	73.30%	67.96%	70.46%	68.11%	67.33%	266618910	0.756
	new-Castle-Lake	24241868	10.8715	79.09%	73.20%	74.44%	74.18%	76.43%	73.33%	77.84%	72.49%	69.16%	70.61%	67.71%	70.41%	263807606	0.748
Penstemon cardwellii	car-Mt-St-Helens	36858317	16.006	78.78%	72.62%	73.08%	74.79%	74.86%	71.02%	78.11%	68.85%	67.87%	71.91%	58.15%	65.45%	259795581	0.736
	car-Siuslaw	28739053	13.0176	76.75%	70.13%	70.98%	72.95%	74.65%	68.79%	76.37%	67.51%	64.66%	69.54%	55.19%	59.94%	252987272	0.717
	car-Tillamook	32770333	13.5502	77.32%	70.70%	71.11%	72.61%	74.83%	68.85%	76.14%	68.68%	64.68%	69.17%	54.63%	60.62%	253803365	0.719
Penstemon fruticosus	fru-Mt-Rainier	21818078	9.9509	77.37%	70.47%	70.00%	73.38%	73.30%	70.13%	76.55%	66.27%	65.62%	65.80%	53.05%	64.85%	252795720	0.716
	fru-var-*praeteritus*	42247127	16.3052	77.71%	71.17%	71%	73.33%	73.58%	70.27%	76.89%	66.96%	67.76%	69.03%	51.65%	69.97%	254698494	0.722
Penstemon lyallii	*P. lyallii*	28008540	12.9174	74.10%	66.74%	64.43%	69.32%	69.11%	64.44%	73.37%	61.40%	60.49%	63.77%	37.92%	57.22%	236938120	0.671
Penstemon montanus	*P. montanus*	28564984	12.2487	72.85%	64.82%	64%	67.48%	69%	63.18%	71.48%	61.70%	59.95%	60.07%	41.67%	56.54%	233235972	0.661
	**Total**	547273439	-	-	-	-	-	-	-	-	-	-	-	-	-	-	-
	**Average**	30404080	12.878	0.785	0.723	0.721	0.741	0.753	0.711	0.775	0.695	0.675	0.698	0.568	0.665	258641220	0.733
	**SD**	5598161.809	1.729	0.024	0.031	0.035	0.025	0.028	0.034	0.025	0.041	0.034	0.036	0.083	0.054	10691775	0.030

After quality control, filtering, and mapping to the *P. davidsonii* reference genome, our whole-genome resequencing efforts produced 547.3 million paired-end reads, for an average of 30.4 ± 5.6 million reads per sample. Genome-wide depth of coverage was fairly consistent across samples (mean: 12.9x ±1.7x). The average per-scaffold coverage was > 70% for most of the eight largest scaffolds, while average coverage for the smaller four scaffolds was slightly reduced (56.8%-69.8%). Genome-wide coverage for most samples was > 70% (mean: 73.3% ±3.0%), with slightly lower coverage for the two outgroup species *P. montanus* and *P. lyallii* (66.1% and 67.1%, respectively).

Table S.3. Anthocyanin extractions from floral tissues.

Species	Sample	Delphinidin	Cyanidin	Pelargonidin
Penstemon rupicola	rup-Castle-Lake			
	rup-Rattlesnake			
	rup-Mt-Rainier	-	+	-
	rup-Cle-Elum	-	+	-
Penstemon davidsonii	dav-Mt-Ashland	+	+	-
	dav-Crater-Lake			
	dav-Paulina-Peak			
Penstemon newberryi	new-Dorado	-	+	-
	new-Plumas	-	+	-
	new-var-*berryi*	+	+	-
	new-Castle-Lake	-	+	-
Penstemon cardwellii	car-Mt-St-Helens	+	-	-
	car-Siuslaw			
	car-Tillamook	+	+	-
Penstemon fruticosus	fru-Mt-Rainier	+	-	-
	fru-var-*praeteritus*			
Penstemon lyallii	*P. lyallii*			
Penstemon montanus	*P. montanus*			

Colored boxes with a "+" symbol indicate the corresponding anthocyanidin was present in the sample. Black boxes indicate samples for which no anthocyanidins were successfully extracted.

Table S.4. Results of all possible ABBA-BABA tests.

# of tests	Bonf. Corr	# significant	% significant
550	9.0909E-05	352	0.64

Tests are not included in the calculation if P1, P2, and P3 all represent the same species.

Table S.5. ABBA-BABA tests for the triplet: car-new-rup

**All samples included**								
**P1**	**P2**	**P3**	**Dstatistic**	**Z-score**	**p-value**	**BBAA**	**ABBA**	**BABA**
car	new	rup	0.00329235	0.825638	0.40901	527300	341400	339159

**Castle Lake samples removed**								
**P1**	**P2**	**P3**	**Dstatistic**	**Z-score**	**p-value**	**BBAA**	**ABBA**	**BABA**
car	new	rup	-0.0367873	12.5607	**2.30E-16**	531508	318122	342422

Top: All samples of *P. newberryi* , *P. cardwellii* , and *P. rupicola* were included. Bottom: The *P. newberryi* and *P. rupicola* samples from Castle Lake were removed.

Table S.6. Regression coefficients between gene density and genetic distance/divergence

50kb windows				10kb windows			
dxy							
species	coeff	r2	pval	species	coeff	r2	pval
car_dav	0.02679961	0.14119495	2.76E-212	car_dav	0.01609035	0.06275877	0
car_fru	0.02636156	0.13185197	1.86E-192	car_fru	0.01584807	0.05928468	0
car_new	0.01537699	0.06985337	8.41E-103	car_new	0.01006378	0.03328736	1.60E-228
car_rup	0.0269757	0.13807176	3.57E-206	car_rup	0.01577318	0.05866475	0
dav_fru	0.02228994	0.1116958	6.06E-158	dav_fru	0.01467893	0.05656848	0
dav_new	0.02946169	0.16153785	2.17E-244	dav_new	0.01740973	0.07200574	0
dav_rup	0.02941799	0.15245021	2.33E-225	dav_rup	0.0176387	0.06949725	0
fru_new	0.02872283	0.1493096	9.15E-227	fru_new	0.01747815	0.0691676	0
fru_rup	0.02864688	0.13858072	2.00E-198	fru_rup	0.01722718	0.06453764	0
new_rup	0.03106926	0.16593077	3.25E-254	new_rup	0.01780928	0.07051457	0
FST							
species	coeff	r2	pval	species	coeff	r2	pval
car_dav	0.55050083	0.22293825	0	car_dav	0.23727683	0.08361573	0
car_fru	0.54103045	0.24688627	0	car_fru	0.23683177	0.09259425	0
car_new	0.38439291	0.20907734	0	car_new	0.16989382	0.06776272	0
car_rup	0.58889745	0.24979735	0	car_rup	0.25661709	0.09767121	0
dav_fru	0.39585108	0.14393333	1.35E-206	dav_fru	0.17170201	0.05242635	4.94E-324
dav_new	0.56023971	0.21803758	0	dav_new	0.23955281	0.08291792	0
dav_rup	0.56416142	0.20889173	2.33E-318	dav_rup	0.24297981	0.08341706	0
fru_new	0.50394301	0.23324547	0	fru_new	0.22201218	0.08579032	0
fru_rup	0.52722745	0.21385034	9.72E-319	fru_rup	0.22923039	0.08534064	0
new_rup	0.58867356	0.24996978	0	new_rup	0.25806691	0.099132	0
π							
species	coeff	r2	pval	species	coeff	r2	pval
car	-0.0026945	0.00338977	2.91E-06	car	0.00102094	0.0004971	6.35E-05
dav	0.0034359	0.00441244	9.44E-08	dav	0.00522008	0.01081117	4.75E-78
fru	0.00287774	0.00296914	1.21E-05	fru	0.00458967	0.0075898	3.38E-55
new	0.00354034	0.00587786	7.17E-10	new	0.0039479	0.00740531	5.55E-54
rup	0.00148633	0.00090986	0.01545792	rup	0.00368041	0.00584709	6.07E-43
